# Fecal matter transplantation and cognition: a systematic review in Alzheimer’s disease and related disorders

**DOI:** 10.1002/alz70859_107684

**Published:** 2025-12-26

**Authors:** Maria Fernanda Peruci Felippe, Isabela Alicia Fink, Laura Carolina Nardi Motta, Matias Pinheiro de Macêdo Neto, Jessica Vargas Lopes, Ana Carolina Gomes Petry, Gabriela Bezerra Sorato, Daniel Pasini Gonçalves, Gabrielle Guindani Maia, Leonardo Bedatti Koehler

**Affiliations:** ^1^ Fundação Universidade Federal de Ciências da Saúde de Porto Alegre, Porto Alegre, Rio Grande do Sul Brazil; ^2^ Universidade Luterana do Brasil, Canoas, Rio Grande do Sul Brazil; ^3^ Universidade Federal do Rio Grande do Sul, Porto Alegre, Rio Grande do Sul Brazil

## Abstract

**Background:**

The gut‐brain axis plays a crucial role in neurodegenerative diseases, such as Alzheimer’s disease (AD). Fecal microbiota transplantation (FMT) has emerged as a potential therapy for AD by restoring microbial balance and reducing neuroinflammation. However, clinical evidence remains limited. This study reviews its potential effects on cognition in AD and other cognitive disorders.

**Method:**

PubMed, Cochrane, Scopus, and Embase databases were searched for human studies on FMT and cognition in AD. Eligible studies included clinical trials, case series, and case reports. Reviews, editorials, animal, and non‐English studies were excluded. Two reviewers screened studies; four extracted data and assessed quality. Meta‐analysis was not performed due to heterogeneity.

**Result:**

Five studies were included, totaling 26 dementia patients, all of whom had recurrent Clostridium difficile infection (CDI). The studies included one randomized controlled trial (RCT, n = 20) and four observational studies (three case reports and one case series, n = 6). FMT was associated with cognitive improvements, particularly in patients with mild cognitive impairment or AD. The RCT demonstrated significant gains in MMSE (MD 6.0, p=0.01) and CDR‐SOB (MD ‐3.1, p=0.048) scores at three months follow‐up. One case series (n=5) reported cognitive improvements post‐FMT, with MMSE increasing from 11 to 17, MoCA from 12 to 21, and CDR‐SOB decreasing from 10 to 5.5. The three case reports described cases of AD exhibiting increased MMSE after FMT (15 to 29, 8 to 13, 5 to 12, respectively). They also noted improvements in mood, social interaction, and performance of daily activities. Beyond cognitive changes, FMT led to gut microbiota modulation, with increased Bacteroidaceae and reduced Enterococcaceae. These microbiome shifts correlated with reduced neuroinflammation and metabolic improvements. Adverse effects were minimal, such as transient nausea and mild abdominal discomfort, with no serious events.

**Conclusion:**

Preliminary evidence suggests FMT may have cognitive benefits in AD patients and recurrent CDI. However, the limited number of studies and the presence of CDI as a common comorbidity highlight the need for larger, controlled trials to better define its role in AD management.

